# Lithium Loss in Vacuum Deposited Thin Films

**DOI:** 10.1021/acsenergylett.4c00153

**Published:** 2024-03-26

**Authors:** Adam J. Lovett, Ahmed Kursumovic, Judith L. MacManus-Driscoll

**Affiliations:** † Department of Materials Science and Metallurgy, University of Cambridge, 27 Charles Babbage Road, Cambridge CB3 0FS, United Kingdom; ‡ Department of Chemical Engineering, University College London, Torrington Place, London, United Kingdom, WC1E 7JE

Thin films serve as ideal model systems for fundamental studies of batteries. Specifically, their very flat surfaces are ideal to probe interfacial phenomena^1−3^ and the ability to grow epitaxial films free of grain boundaries enables the study of orientational-dependent electrochemical properties.^4−9^ For a wide range of systems, physical vapor deposition (PVD) techniques are the methods of choice for thin film growth, particularly magnetron sputtering for its high-area capabilities which have led to commercialization,^10^ and pulsed laser deposition (PLD) for exploratory science owing to its relative ease and potential for stoichiometric transfer of material from target to substrate.^11^ However, stoichiometric transfer is not guaranteed for volatile systems, particularly for materials containing lithium. Thus, small changes in the process parameters leads to drastic differences in film composition,^12,13^ phase purity (lithium deficient phases are often observed in PVD films^5,14−16^), and consequent film properties.^17,18^ Yet, for battery and lithionic applications which utilize thin films, particularly solid-state electrolytes, optimal stoichiometric composition is essential for good electrochemical performance.^19−21^

Lithium loss during deposition is unavoidable. In fact, it has been both theoretically predicted^22^ and experimentally observed for vacuum deposition methods. Figure 1a highlights some of the lithium loss mechanisms that occur during vacuum deposition (including during the target preparation).^12,13,23^ Notable loss mechanisms are (1) thermal evaporation due to lithium being very volatile, resulting in losses in both the film and target when exposed to high temperatures, (2) high atmospheric sensitivity of the grown films when exposed to ambient air leading to loss of lithium from the film due to the formation of degradation products, and (3) scattering of Li/Li-oxide species in the plasma plume during deposition at higher gas partial pressures resulting in lithium deficiency in the growing film. Analytical modeling (Figure 1b,c) and experimental evidence (Figure 1d) of scattering has been measured in lithium-containing films.^22,24^ Lithium has a high scattering probability due to it being lighter than both the background gas (typically O_2_) and the other species in the plume (often transition metals with a significantly higher mass). This makes it particularly prone to backscattering, resulting in a wide apex angle plume and a very small area where high Li content is achievable. If this area is smaller than the substrate, or misaligned, homogeneous films cannot be prepared and composition stray will occur across the film. Further, due to the high velocity of the incoming lithium species (evidenced by the faster expansion of plume in Figure 1c), they can rebound from the surface of the substrate and so not be incorporated in the film, resulting in further deficiency.^13,24^

**Figure 1:**
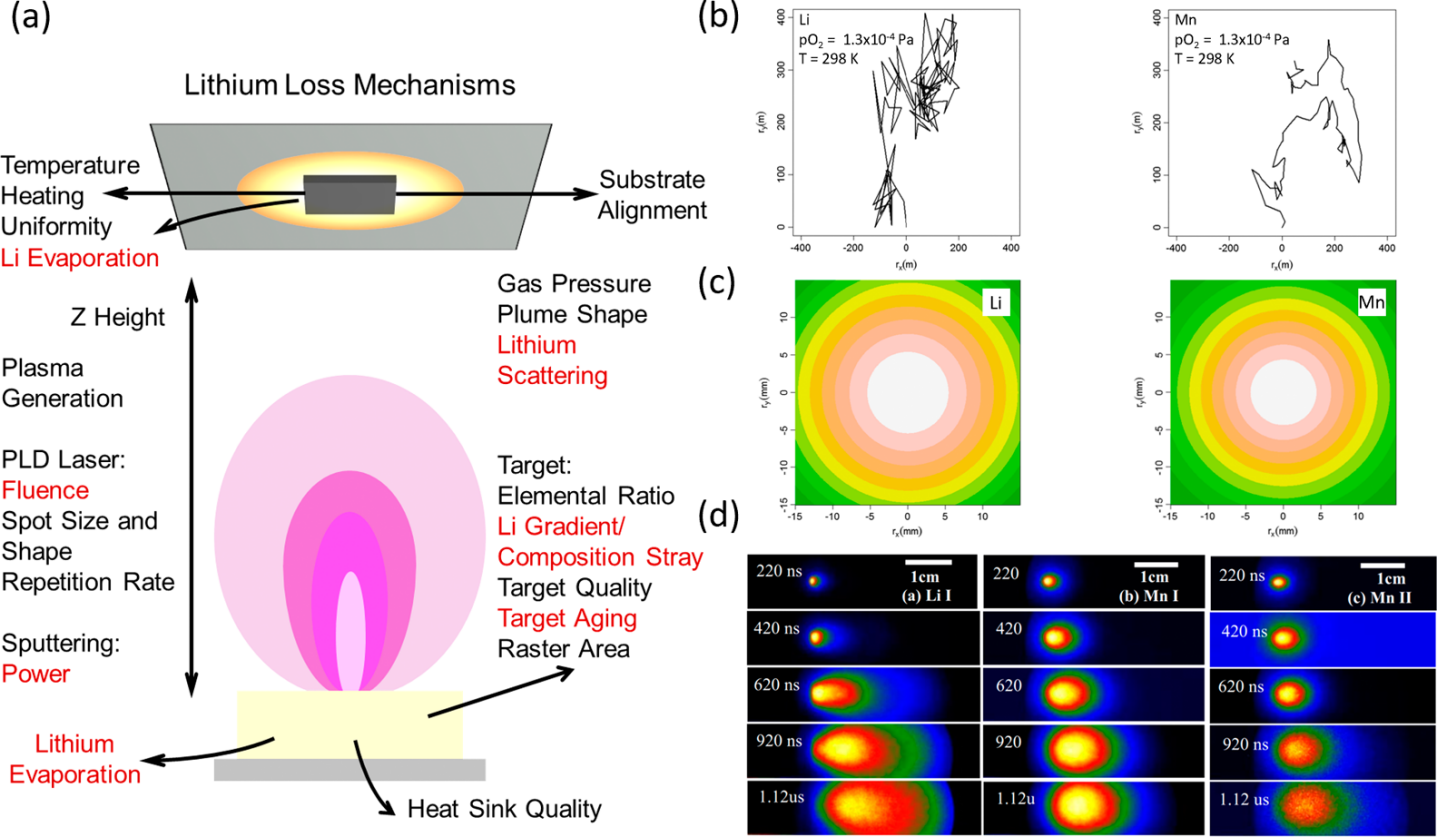
Origins of lithium loss during vacuum deposition. (a) Schematic of plasma generated during vacuum deposition highlighting process parameters and mechanisms that can influence the lithium stoichiometry of the thin film. Processes where direct loss of lithium can occur are highlighted in red. (b) and (c) Theoretical and (d) experimental evidence of lithium scattering during pulsed laser deposition (PLD) of LiMn_2_O_4_ films. (b) Trajectory simulations of stochastic scattering of Li and Mn at pO_2_ = 10^–6^ Torr and *T* = 298 K. Lithium is scattered more violently than manganese and undergoes significant backscattering, constituting to lithium deficiency. (c) Simulated probability density plots for Li and Mn during PLD (pO_2_ = 1.3 Pa, *T* = 298 K, expansion after 5 μs with v = 10^2^ m s^–1^, a typical average velocity for ablated atoms^58^ (0.05 mm Z height)). Li scatters over a wider area than Mn. Reproduced with permission from ref (22). Copyright 2013 American Physical Society. (d) Space and time-resolved emission spectroscopy of a LiMn_2_O_4_ PLD plume. The plume dynamics are clearly different for Li and Mn, with increased angular broadening of the plume observed for Li I. This is a direct consequence of scattering and demonstrates the importance of optimizing the deposition pressure and Z height. Reproduced with permission from ref (24). Copyright 2009 AIP Publishing.

While Figure 1 predominantly focuses on PLD, it should also be noted that these loss mechanisms are equally relevant to other vacuum deposition techniques such as sputtering. Sources of heat will result in thermal loss mechanisms and the presence of gas collisions (e.g., with O_2_, N_2_, Ar, etc.) will cause a depletion of species in the films due to gas scattering. Also, exposure to the atmosphere (primarily H_2_O and O_2_) will lead to degradation product formation on the film surface.

In fact, lithium is not the only element prone to compositional stray. For example, SrRuO_3_, Sr_3_Ru_2_O_7_, and Sr_2_RuO_4_ thin films can be grown from the same stoichiometric SrRuO_3_ target merely by fine-tuning the PLD processing parameters to account for the volatility of ruthenium.^18^ Moreover, to grow stoichiometric BiFeO_3_ excess bismuth is often added to targets to compensate for bismuth volatility.^25−27^ Additionally, sodium excess is often added to Na-containing films (e.g., Na_*x*_CoO_2_ and Na_*x*_MnO_2_) to account for significant Na vaporization.^28−30^ Unlike the aforementioned examples, the challenge for lithium-containing films is that loss occurs through more than one mechanism. Lithium is both very volatile and very prone to gas scattering. Adding excess lithium to the target does not necessarily result in stoichiometric films. Consequently, careful optimization of the process parameters is required in order to grow high-quality stoichiometric films free from lithium-deficient impurity phases, particularly the target stoichiometry (not necessarily the desired film composition) and substrate positioning in the plume.^12,13,23^ Additionally, there are more nuanced parameters/phenomena specific to the given PVD technique that must be considered. See the following reviews that cover these aspects in detail: PLD,^12,13,23^ sputtering^31,32^ and molecular beam epitaxy.^33^

Lithium loss manifests differently for different phases, examples of which are presented in Figure 2. For LiCoO_2_ (LCO) lithium loss results in the formation of Co_3_O_4_ (Figure 2a,b), which does not lithiate in the LCO voltage window (3.7–4.2 V).^34^ Therefore, LCO films with significant Co_3_O_4_ impurities will exhibit diminished areal/specific discharge capacities.^19,35^ Further, lowered lithium content in LCO can lead to the formation of nonstoichiometric two-phase (hexagonal and monoclinic) films, as detected during electrochemical testing (Figure 2c).^36^ This can impair electrochemical performance, due to nonuniform volume changes causing microcracking, which results in increased capacity fade.^37^ For LiMn_2_O_4_ (LMO), Mn_2_O_3_ and Mn_3_O_4_ are often detected (Figure 2d).^5,38−40^ Again, films with high MnO_*x*_ content will exhibit lowered areal/specific discharge capacities. Epitaxial films of different orientation show different tendencies to form manganese oxide impurity phases, with the (001) orientation being more prone ascribed to preferential alignment of the fast Li^+^ conduction channels abetting volatility.^5^ If the growth temperature is too high (>∼650 °C), lithium can be completely driven off resulting in the spinel LMO phase not forming.^41^ Conversely, if the growth temperature is significantly lowered, this can result in the formation of lithium-rich LMO, including Li_2_Mn_2_O_4,_ compensating for volatility issues.^42^

**Figure 2:**
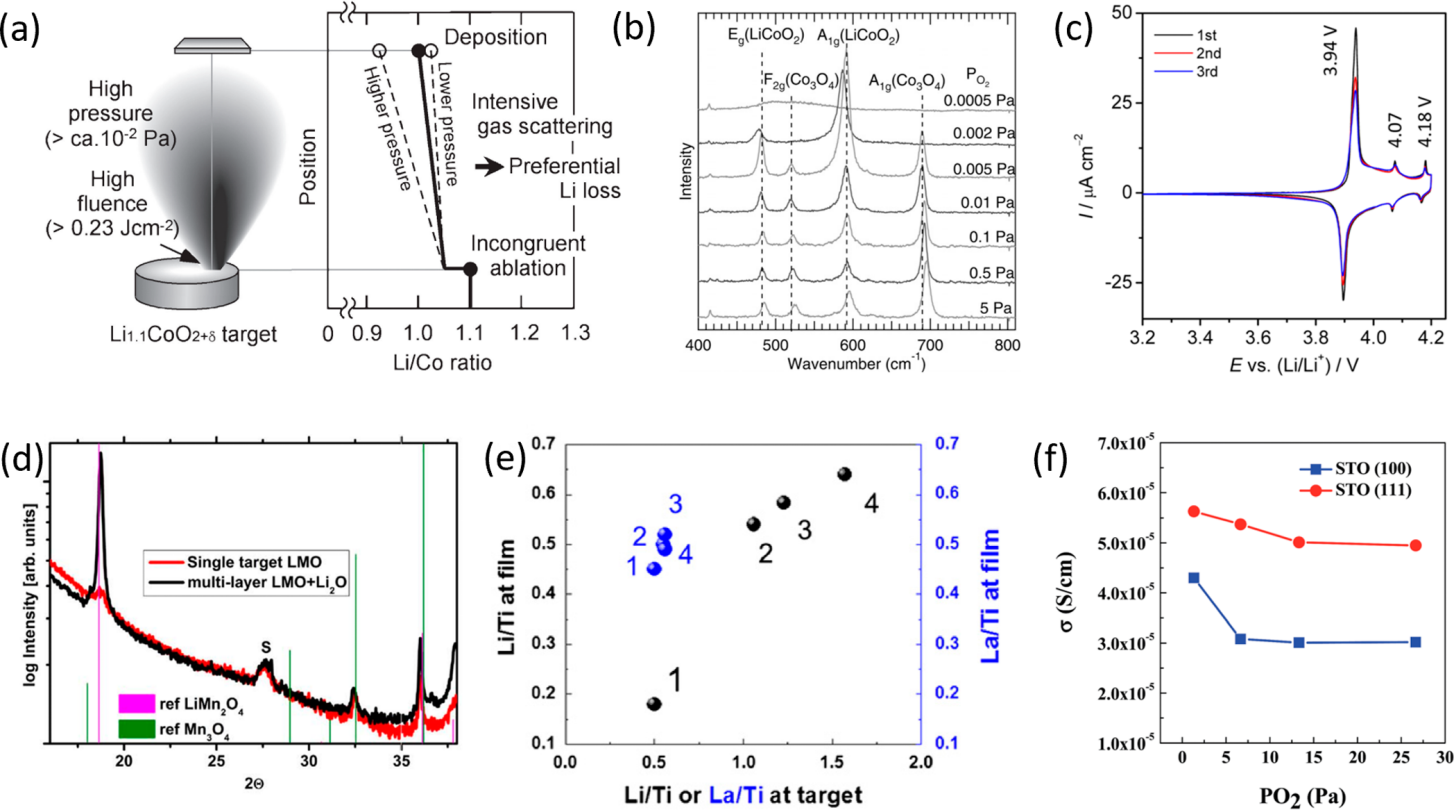
Macroscale evidence of lithium loss in PLD battery thin films. (a) Schematic and (b) Raman spectroscopy evidence of compositional deviation in Li-rich LiCoO_2_ target. Increasing the pO_2_ during growth intensifies Li gas scattering, resulting in an enhanced Co_3_O_4_ Raman signal. Reproduced with permission from ref (35), copyright 2012 IOP Publishing, and ref (19), copyright 2010 Springer Nature. (c) Cyclic voltammogram of LCO/SRO/STO (111) film cycled with an upper cutoff voltage of 4.2 V (corresponding to Li_*x*_CoO_2_, *x* > 0.5). Three redox peaks are present: the major 3.94 V peak corresponds to a first-order metal–insulator transition, and the two minor peaks correspond to the order–disorder transitions around Li_0.5_CoO_2_.^59,60^ Observation of the minor redox peaks in the first cycle is indicative of the presence of LCO, with *x* < 0.5. Reproduced with permission from ref (21). Copyright 2015 American Chemical Society. (d) XRD pattern of LMO thin films, with reflections marked in green corresponding to Li deficient impurity phase Mn_3_O_4_. Reproduced with permission from ref (40). Copyright 2018 Elsevier. (e) Measured composition and (f) ionic conductivity of LLTO thin films. In (e), the Li/Ti ratio of the target is not retained in the film, even when significant excess of Li is added. This impacts the measured ionic conductivity (f). First, all films exhibit 2 orders of magnitude lower ionic conductivity than bulk (10^–3^ S cm^–1^).^61^ Further reduction in conductivity occurs with increasing pO_2_ due to Li scattering. Reproduced from ref (36) with permission. Copyright 2016 Royal Society of Chemistry.

For the solid-state electrolytes Li_3*x*_La_2/3–*x*_TiO_3_ (LLTO) and Li_7_La_3_Zr_2_O_12_ (LLZO), lowered Li^+^ ionic conductivity, typically 2–3 orders of magnitude lower in thin films vs bulk, is often reported and ascribed to lithium loss (Figure 2e,f).^36,43,44^ The reduced conductivity arises due to a net loss of Li^+^ charge carriers during deposition, exacerbated by volatile loss mechanisms due to the very high growth temperatures required for high crystallinity (often >800 °C). Further, Li deficient phases are also often detected in LLTO and LLZO, which likely impede performance. TiO_2_,^14,45,46^ La_2_Ti_2_O_7_,^14,45,47^ and spinel Li_4_Ti_5_O_12_^6,47^ are commonly reported in LLTO films. For LLZO, the Li-deficient phase La_2_Zr_2_O_7_ is often observed, impacting Li^+^ transport, reducing the ionic conductivity and raising the activation energy.^12,15,48^ Further, the high reactivity of LLZO toward CO_2_ leads to Li_2_CO_3_ formation on the film surface, increasing the interfacial resistance when implimented in solid-state batteries.^43^ It should also be noted that Li-deficient impurity phases are often detected in thicker films (>100 nm) but are less common in thinner films (<100 nm). Li-deficient impurity phases may be equally prevalent in thinner films owing to the difficulty in resolving X-ray diffraction (XRD) reflections in thinner films.^6^

A number of approaches have been employed to compensate for lithium loss in thin films during deposition including: adding excess lithium to the PLD target, typically between 5 and 20 wt % of lithium;^5,6,14^ growth of superlattice films with alternating layers of the phase to be studied and a lithium-rich phase, e.g., Li_2_O or Li_3_N, to supply lithium to the film of interest;^44,49^ codepositing with a lithium-rich phase;^50^ or post-annealing in lithium-rich atmospheres to reintroduce lithium to the as-grown film.^15^ While these approaches help maintain higher lithium content in the said films, there is no fine control of the lithium incorporation, and in some cases the approaches produce unwanted microstructural defects, such as voids within the superlattice film due to collapse of the sacrificial lithium-rich layers.^51^ Therefore, there is still no guarantee that either the targeted lithium stoichiometry is achieved or that unwanted secondary phases will not form. In fact, Li-deficient MnO_*x*_ phases are detected in LMO thin films with 100 wt % excess of Li.^5^ Thus, if the film stoichiometry cannot be carefully controlled, the electrochemical performance will vary from point to point across the sample, which is potentially detrimental to the overall performance of the film.

As highlighted, the majority of studies focus on macroscale (thus the average) composition of the film (Figure 2). However, normally macroscopic in-plane measurements are made using large area (>1 mm^2^) square/bar electrodes and so average (rather than local) film properties are measured.^6,14,15,20,44^ Hence, there is no information on the variation of properties across the film area. Considering the high potential for lithium variability across Li-based thin films, it is important to be able to understand the local properties point-to-point across a sample. This is particularly important when long-range homogeneous performance is vital, such as lithionic devices for neuromorphic computing.

Instead, we utilize the microdot approach to measure the *local* Li^+^ ionic conductivity (out-of-plane geometry) across the total area of a Li_*x*_La_0.32_Nb_0.7_Ti_0.3_O_3_-based (LL(Nb,Ti)O) film (22 discrete gold microdot electrodes with 0.07 mm^2^ area). This system was chosen for its sizable Li^+^ ion conductivity, up to 10^–4^ S cm^–1^ at 25 °C.^52^ Please refer to Supporting Information Note 1 for an extended discussion. Across the film, a 2 orders of magnitude distribution in Li^+^ ion conductivity is observed (10^–6^–10^–4^ S cm^–1^, Figure 3a). Considering the spatial distribution of the electrodes (Figure 3b), we note that the highest ionic conductivities tend to be observed at the center of the film, whereas lower ionic conductivities are located at the peripheries. The large variations in ionic conductivity across the film are understood by considering how the PLD parameters and plume dynamics influence lithium stoichiometry (recall Figure 1). Specifically, lithium is more strongly gas-scattered than heavier species (Ti, La, and Nb), resulting in deviations from the stoichiometric ratio in a concentric way from the center of the PLD plume,^22,24^ as discussed earlier. Further, titanium is also prone to severe gas scattering, which can result in deviations from the desired Ti:La ratio.^6^ It is noted that, for LLTO, there is a strong relationship between ionic conductivity and lithium content, displaying a parabola-type relationship with a maxima in ionic conductivity (∼10^–3^ S cm^–1^) at Li_3*x*_La_2/3–*x*_TiO_3_, *x* = 0.067.^53,54^ This parabola-type trend is also observed in doped LLTO.^55,56^ The ionic conductivity can deviate by 1–2 orders of magnitude with a small deviations in the composition.^53^

**Figure 3:**
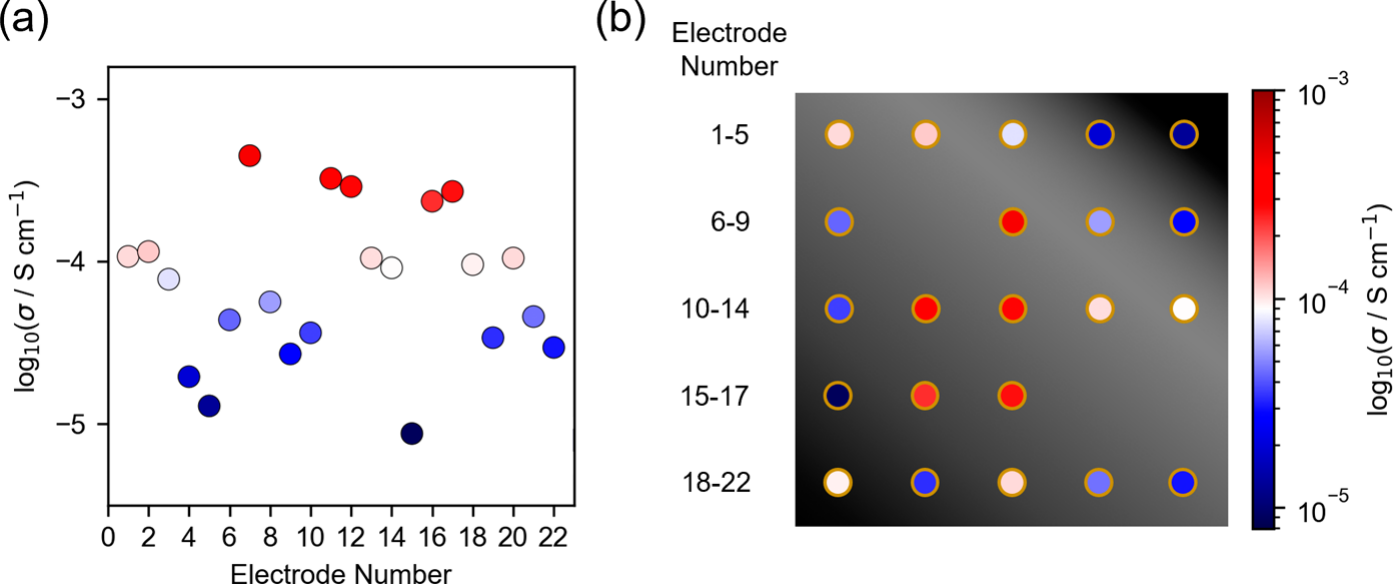
Microscale evidence of lithium loss studied with local ionic conductivity measurements. (a) magnitude of Li^+^ ionic conductivities calculated from 22 gold microdot electrodes (300 μm in diameter). (b) Spatial location of the electrodes where the colors corresponds to the magnitude of ionic conductivity. See the Supporting Information for an extended figure.

It should be stressed that the observations in Figure 3 are not unexpected. Our work highlights that this phenomenon can occur on a *local* scale, point-to-point across the sample, and that lithium-containing films are particularly prone to deviations. This has potentially detrimental consequences for the electrochemical performance of the film which warrants consideration.

We emphasize here that, like many other studies,^5,6,14^ we added excess lithium to our PLD target to combat lithium loss (10 wt % excess Li_2_O). And yet, through spatially mapping the ionic conductivity of our film we observe clear inhomogeneity in ionic conductivity across the sample (Figure 3). Our results have implications for the wider thin film battery and lithionics fields. First, our results demonstrate that adding excess lithium to PLD targets does not necessarily eliminate lithium loss on a *local* scale. Hence, sample inhomogeneity may be an intrinsic property not accounted for in many lithium-containing PLD films. Second, macroscale electrochemical impedance spectroscopy (EIS) measurements utilizing bar electrodes that measure the *average* performance by probing large regions of the sample (e.g., using bar electrodes) disguise *local* inhomogeneities. This may account for why many PLD grown LLTO planar films exhibit lower-than-expected Li^+^ ionic conductivity.^6,14,57^ Finally, our results validate the need to map the local properties of lithium-containing thin films, so that “*more averaged*” measurements can be contextualized. This is particularly relevant when comparative macroscale measurements are undertaken (i.e., measuring the properties of a film exposed to a stimulus and comparing it with a reference sample) such that inhomogeneities are accounted for. Crucially, a greater understanding of the film on a *local* scale will improve the macroscale performance and thus overall quality of the resultant film.

As a final remark, it is worth considering when and where lithium loss poses the most significant problems. With a well-optimized PVD growth, it is possible to mitigate against lithium loss entirely, the complexity of which ultimately depends on the technique chosen and the desired characteristics of the film. For example, an amorphous/polycrystalline film of a tertiary system (e.g., Li, O + 1 other element) grown at low/room temperature is less likely to be impacted by lithium loss than an epitaxial complex oxide containing many elements grown at high temperature (>500 °C). Naturally, there will be film-systems/PVD growth conditions where lithium loss is not as significant. Nonetheless, the challenges highlighted within our viewpoint still need to be considered and overcome. Finally, the material grown and its intended purpose will also factor into the impact that lithium loss has. For batteries, negative electrodes are unlikely to present problems, as the majority are grown in their delithiated state (e.g., Si, C, TiO_2_, Nb_2_O_5_, etc.).^12^ Solid electrolytes are particularly prone as the loss of charge carriers will result in lowered ionic conductivity and a tendency to form Li-deficient impurity phases. Both are very detrimental to performance and will lead to inhomogeneous performance, thus careful optimization of processing parameters is essential, particularly the target composition (not necessarily the desired film composition) and the substrate positioning in the plume.^13^ For positive electrodes such as LiMn_2_O_4_, LiCoO_2_, and LiNi_1–*x*–*y*_Mn_*x*_Co_*y*_O_2_ (NMC) (and lithium-containing negative electrodes like Li_4_Ti_5_O_12_), it really depends on the cycling conditions and final application. If the film is to be cycled in an environment where excess lithium is present, e.g., half-cell vs Li metal in liquid electrolyte, with suitable voltage cut-offs that enable full lithiation, low-level lithium deficiencies are unlikely to be detrimental provided it does not hinder the formation of the phase. This is because the film can relithiate during cycling. However, if lithium cannot be reintroduced to the film, or there is a small lithium reserve (e.g., in a solid-state battery), then lithium loss can pose significant performance limitations due to Li-deficient phase formation, strongly limiting the achievable current density and total areal/specific capacity of the film.

To summarize, from a survey of current literature, this Viewpoint identifies and discusses lithium loss mechanisms during vacuum deposition methods. Next, by spatially mapping variations in Li^+^ ionic conductivity across LL(Nb,Ti)O-based thin films, we found the Li^+^ ionic conductivity varies by 2 orders of magnitude across the total film area (25 mm^2^). This large variation is attributed to severe gas scattering of light lithium species during deposition. This result demonstrates that large variable local performance is present, an intrinsic property in many films which will not be detected by commonly undertaken macroscale measurements. This work highlights the need to investigate local performance variation arising from lithium nonstoichiometry which is prevalent in vacuum grown thin films.

## Supporting Information Available

Experimental section and extended discussion of spatial mapping of Li^+^ ionic conductivity in LL(Nb,Ti)O-based thin film (microstructure, schematic and cross-sectional TEM; EIS, Nyquist plots) (PDF)
