# Supplementary material for: Lithium Loss in Vacuum Deposited Thin Films
Source: ACS Energy Lett. 2024 Mar 26;9(4):1753–8. doi: 10.1021/acsenergylett.4c00153 (PMC11019639; doi:10.1021/acsenergylett.4c00153)
Supplement: Supplementary file 1 — nz4c00153_si_001.pdf [file nz4c00153_si_001.pdf]

Supporting Information: Lithium Loss in Vacuum Deposited Thin Films

Author Names: Adam J. Lovett*, Ahmed Kursumovic, and Judith L. MacManus-Driscoll
Affiliation(s):
[1]Department of Materials Science and Metallurgy, University of Cambridge, 27 Charles Babbage Road, Cambridge CB3 0FS, United Kingdom
[2] Department of Chemical Engineering, University College London, Torrington Place, London, United Kingdom, WC1E 7JE
*Corresponding Author E-mail Address ajl207@cam.ac.uk/adam.lovett@ucl.ac.uk

Experimental

Film Composition Studied: We grow and study a model system in which there is sizeable Li^+ ion conductivity, a $Li_xLa_{0.32\pm0.05}(Nb_{0.7\pm0.1}Ti_{0.32\pm0.05})O_{3\pm\delta}$-$Ti_{0.8\pm0.1}Nb_{0.17\pm0.03}O_{2\pm\delta}$-anatase vertically aligned nanocomposite (VAN) epitaxial thin film system (herein referred to as LL(Nb,Ti)O-(Ti,Nb)O_2) with a thickness of 100 nm. We have previously reported this system [1]. In this VAN film system, (Ti,Nb)O_2 nanocolumns (~20-50 nm in diameter) are embedded in a highly Li^+ ion conducting (> 10^{-4} S cm^{-1} at 25 °C) LL(Nb,Ti)O matrix. The electronic conductivity through the (Ti,Nb)O_2 nanocolumns is negligible with respect to the lithium ionic conductivity due to the presence of a rectifying junction (see Ref [1]). A schematic and TEM image of the LL(Nb,Ti)O-(Ti,Nb)O_2 VAN is shown in Figure S1a.

Target Preparation and Pulsed Laser Deposition of Films: Targets and PLD methodologies are reported in our previous publication [1]. The PLD target contains a 10 % wt. excess of Li_2O. Highly crystalline and epitaxial VAN films grown on (001) oriented Nb-doped STO (Nb-STO) (0.5% wt. Nb) substrates were achieved with the following growth conditions: T_{sub} = 880 °C, F = 1.0 J.cm^{-2}, pO_2 = 10Pa, ν = 8Hz, substrate-target distance = 45 mm.

Electrical Impedance Spectroscopy: Out-of-plane (top to bottom) electrical impedance spectroscopy (EIS) measurements were performed on 100 nm thick films grown on electronically conducting Nb-STO. A Biologic SP200 potentiostat was used to measure samples with a 50mV applied AC voltage between 10Hz - 1MHz. The samples were mounted on a thin glass slip with conductive silver paste, which also acted as the bottom electrode, and connected to a LinkAm HFS350 heating stage fitted with probe arms. Gold top dot electrodes 300µm in diameter were deposited by RF sputtering using a shadow mask.

Supporting Information Note 1: Extended Results and Discussion of Figure 3

For electrolytes, an important metric is the Li^+ ion conductivity (σ). However, normally macroscopic in-plane measurements are made using large area (> 1 mm^2) square/bar electrodes and so average (rather than local) film properties are measured [2–6]. Hence, information is then not gained on the variation of properties across the film area. By utilizing microdot electrodes, that is circular electrode pads with micron dimensions applied to the surface of the film, in

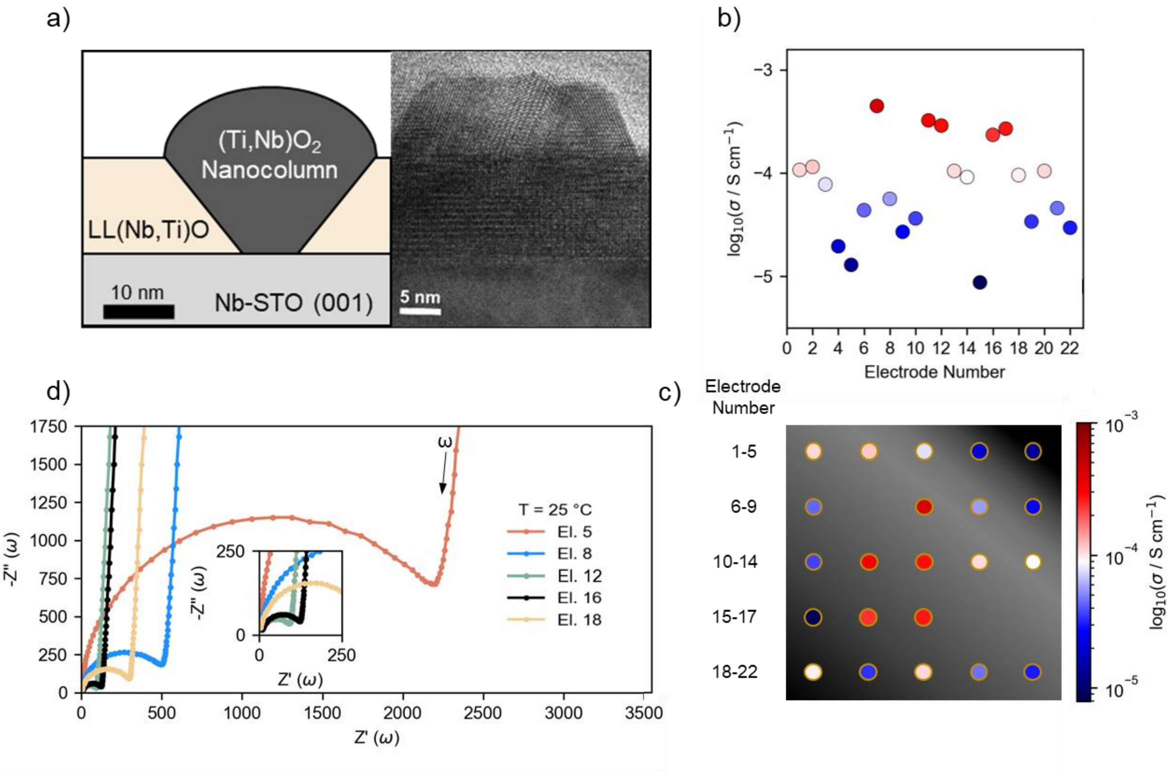

Figure S1: Microscale evidence of lithium loss studied with microscopic ionic conductivity measurements. (a) Schematic of LL(Nb,Ti)O-(Ti,Nb)O_2 VAN film explored, and cross sectional TEM image of the actual microstructure (reproduced from our previous publication)[1]. (b-d) EIS data collected on 22 gold microdot electrodes (300 um in diameter) (b) the magnitude of their ionic conductivities (c) spatial location of the electrodes where the colours corresponds to the magnitude of ionic conductivity (d) Nyquist plots of selected electrodes (El. #) with zoomed in region inset, showing clear variation in ionic conductivity for different electrodes across the film.

combination with an electronically conducting electrode (either the substrate or a buffer layer), *local* properties can be investigated via many out-of-plane measurements [7]. Such microdot electrodes have seen wide use in characterizing the electronic properties of epitaxial thin films, but are typically used to probe only a single point in a sample [1,8–10].

We use the microdot approach to measure *local* Li^+ ionic conductivity across the total film area (22 discrete gold microdot electrodes with 0.07 mm^2 area). We undertake electrochemical impedance spectroscopy (EIS) in an out-of-plane geometry on epitaxial vertically aligned nanocomposite (VAN) LL(Nb,Ti)O-(Ti,Nb)O_2 films at room temperature. This system was chosen for its sizable Li^+ ion conductivity, up to 10^{-4} S cm^{-1} at 25 °C [1]. The films are composed of a matrix of LL(Nb,Ti)O and discrete pillars of (Ti,Nb)O_2 (Figure S1a). Each electrode probes ~0.3 % of the total thin film area in a discrete region. Hence, this spatial EIS approach probes smaller regions than typical square/bar electrodes (> 1 mm^2 areas) [2–6], thus allowing the electrochemical properties to be mapped in a simple way across the total film area (25 mm^2).

The results from our EIS measurements conducted on the 22 electrodes are presented in Figure S1. A two order of magnitude distribution in the Li^+ ion conductivity is observed (10^{-6}-10^{-4} S cm^{-1}, Figure S1b). Considering the spatial distribution of the electrodes (Figure S1c), we note

that the highest ionic conductivities tend to be observed at the center of the film, whereas lower ionic conductivities are located at the peripheries. Nyquist plots (Figure S1d) of selected microdot electrodes clearly show the same features: a single semi-circle arc at a high frequency ascribed to the Li^+-ionic motion and a capacitive response at low frequency indicative of lithium-blocking electrode behavior [1,2]. The variation in high-frequency arc size for different points indicates the wide variation of ionic conductivity across the film.

The large variations in ionic conductivity across the film are understood by considering how the PLD parameters and plume dynamics influence lithium stoichiometry (Figure 1). Specifically, lithium is more strongly scattered by O_2 molecules than heavier species e.g., Ti, La and Nb (recall Figure 1). This results in deviations from the stoichiometric ratio in a concentric way from the center of the PLD plume[11,12], as discussed earlier. Further, titanium is also prone to severe gas scattering, which can result in deviations from the desired Ti:La ratio[2]. It is noted that for LLTO, there is a strong relationship between ionic conductivity and lithium content, displaying a parabola-type relationship with a maxima in ionic conductivity (~ 10^{-3} S cm^{-1}) at $Li_{3x}La_{2/3-x}TiO_3$ x = 0.067 [13,14]. This parabola-type trend is also observed in doped LLTO [15,16]. The ionic conductivity can deviate by 1-2 orders of magnitude with a small deviations in the composition [13]. The radial behavior of compositional stray during deposition [11] is broadly what we observe in our films (Figure S1c). The highest ionic conductivities are observed towards the center because this is where the lithium content is higher. It should be stressed that the observations in Figure S1 are not unexpected. Our work highlights that this phenomenon can occur on a *local* scale, point-to-point across the sample, and that lithium-containing films are particularly prone to deviations. This has potentially detrimental consequences for the electrochemical performance of the film which warrants consideration.

References

(1) Lovett, A. J.; Kursumovic, A.; Dutton, S.; Qi, Z.; He, Z.; Wang, H.; MacManus-Driscoll, J. L. Lithium-Based Vertically Aligned Nancomposite Films Incorporating LixLa0.32(Nb0.7Ti0.32)O3electrolyte with High Li+ion Conductivity. *APL Mater.* **2022**, *10* (5), 0–7. https://doi.org/10.1063/5.0086844.

(2) Ohnishi, T.; Mitsuishi, K.; Nishio, K.; Takada, K. Epitaxy of Li3 x La2/3--x TiO3 Films and the Influence of La Ordering on Li-Ion Conduction. *Chem. Mater.* **2015**, *27* (4), 1233–1241.

(3) Aguesse, F.; Roddatis, V.; Roqueta, J.; Garcia, P.; Pergolesi, D.; Santiso, J.; Kilner, J. A. Microstructure and Ionic Conductivity of LLTO Thin Films: Influence of Different Substrates and Excess Lithium in the Target. *Solid State Ionics* **2015**, *272*, 1–8.

(4) Rawlence, M.; Garbayo, I.; Buecheler, S.; Rupp, J. L. M. On the Chemical Stability of Post-Lithiated Garnet Al-Stabilized Li7La3Zr2O12 Solid State Electrolyte Thin Films. *Nanoscale* **2016**, *8* (31), 14746–14753. https://doi.org/10.1039/c6nr04162k.

(5) Ohnishi, T.; Takada, K. Synthesis and Orientation Control of Li-Ion Conducting Epitaxial Li0. 33La0. 56TiO3 Solid Electrolyte Thin Films by Pulsed Laser Deposition. *Solid State Ionics* **2012**, *228*, 80–82.

(6) Pfenninger, R.; Struzik, M.; Garbayo, I.; Stilp, E.; Rupp, J. L. M. A Low Ride on

Processing Temperature for Fast Lithium Conduction in Garnet Solid-State Battery Films. *Nat. Energy* **2019**, *4* (6), 475–483. https://doi.org/10.1038/s41560-019-0384-4.

(7) Huang, R.; Kucharczyk, C. J.; Liang, Y.; Zhang, X.; Takeuchi, I.; Haile, S. M. Out-of-Plane Ionic Conductivity Measurement Configuration for High-Throughput Experiments. *ACS Comb. Sci.* **2018**, *20* (7), 443–450. https://doi.org/10.1021/acscombsci.8b00037.

(8) Lovett, A. J.; Wells, M. P.; He, Z.; Lu, J.; Wang, H.; MacManus-Driscoll, J. L. High Ionic Conductivity in Fluorite δ-Bismuth Oxide-Based Vertically Aligned Nanocomposite Thin Films. *J. Mater. Chem. A* **2022**, 3478–3484. https://doi.org/10.1039/d1ta07308g.

(9) Lee, S.; Zhang, W.; Khatkhatay, F.; Wang, H.; Jia, Q.; MacManus-Driscoll, J. L. Ionic Conductivity Increased by Two Orders of Magnitude in Micrometer-Thick Vertical Yttria-Stabilized ZrO2 Nanocomposite Films. *Nano Lett.* **2015**, *15* (11), 7362–7369.

(10) Lee, S.; Zhang, W.; Khatkhatay, F.; Jia, Q.; Wang, H.; Macmanus-Driscoll, J. L. Strain Tuning and Strong Enhancement of Ionic Conductivity in SrZrO3-RE2O3 (RE = Sm, Eu, Gd, Dy, and Er) Nanocomposite Films. *Adv. Funct. Mater.* **2015**, *25* (27), 4328–4333. https://doi.org/10.1002/adfm.201404420.

(11) Packwood, D. M.; Shiraki, S.; Hitosugi, T. Effects of Atomic Collisions on the Stoichiometry of Thin Films Prepared by Pulsed Laser Deposition. *Phys. Rev. Lett.* **2013**, *111* (3), 1–5. https://doi.org/10.1103/PhysRevLett.111.036101.

(12) Canulescu, S.; Papadopoulou, E. L.; Anglos, D.; Lippert, T.; Schneider, C. W.; Wokaun, A. Mechanisms of the Laser Plume Expansion during the Ablation of LiMn 2O4. *J. Appl. Phys.* **2009**, *105* (6). https://doi.org/10.1063/1.3095687.

(13) Stramare, S.; Thangadurai, V.; Weppner, W. Lithium Lanthanum Titanates: A Review. *Chem. Mater.* **2003**, *15* (21), 3974–3990.

(14) Sun, Y.; Guan, P.; Liu, Y.; Xu, H.; Li, S.; Chu, D. Recent Progress in Lithium Lanthanum Titanate Electrolyte towards All Solid-State Lithium Ion Secondary Battery. *Crit. Rev. Solid State Mater. Sci.* **2019**, *44* (4), 265–282. https://doi.org/10.1080/10408436.2018.1485551.

(15) Morata-Orrantia, A.; García-Martín, S.; Alario-Franco, M. Á. Optimization of Lithium Conductivity in La/Li Titanates. *Chem. Mater.* **2003**, *15* (21), 3991–3995. https://doi.org/10.1021/cm0300563.

(16) Latie, L.; Villeneuve, G.; Conte, D.; Le Flem, G. Ionic Conductivity of Oxides with General Formula LixLn1/3Nb1- XTixO3 (Ln= La, Nd). *J. Solid State Chem.* **1984**, *51* (3), 293–299.
